# The presence of the wAnD strain of Wolbachia is correlated with lower levels of Plasmodium sporozoites and a less diverse microbiome in wild Anopheles demeilloni mosquito cephalothoraxes

**DOI:** 10.12688/gatesopenres.16384.1

**Published:** 2026-05-15

**Authors:** Seynabou Sougoufara, Janvier Bandibabone, Laura Chatterley, Isabel Hughes, Carolina Molina Taberes, Jade Ball, Thomas Palliaser, Vishaal Dhokiya, Eva Heinz, Grant Hughes, Thomas Walker

**Affiliations:** 1 University of Warwick, Coventry, UK; 2 CRSN, Lwiro, Sud-Kivu, Democratic Republic of the Congo; 3 University of Strathclyde Institute of Pharmacy and Biomedical Sciences, Glasgow, Scotland, UK; 4 University of Leicester, Leicester, UK; 5 Liverpool School of Tropical Medicine, Liverpool, England, UK

**Keywords:** Anopheles demeilloni, Wolbachia, wAnD strain, Plasmodium falciparum, microbiome

## Abstract

**Background:**

The increasing insecticide resistance of malaria vectors is an urgent concern for disease control and novel vector control strategies are needed. *Wolbachia* are endosymbiotic bacteria that can invade mosquito populations and reduce transmission of human pathogens. *Wolbachia* strains in wild *Anopheles (An.)* malaria vectors are rare, with only two known genuine symbioses; *An. moucheti* with *w*AnM and *An. demeilloni* with *w*AnD. In this study, we set out to determine if there was a correlation between *w*AnD in different *An. demeilloni* mosquito body parts, infective stage *Plasmodium (Pl.) falciparum* malaria sporozoites in cephalothoraxes and the mosquito microbiome.

**Methods:**

We undertook a combination of quantitative PCR, *16S rRNA* amplicon sequencing and sanger sequencing of the *Wolbachia surface protein* ( *wsp*) gene after isolating *An. demeilloni* female body parts from wild caught individuals collected in 2021 and 2024 from the Sud Kivu region of Democratic Republic of Congo. Results *Wolbachia* prevalence rates were significantly higher in abdomens compared to cephalothoraxes and density was also significantly higher in abdomens (P<0.0001). Overall sporozoite prevalence was 1.3% (9/704) which was not significantly different between *Wolbachia*-positive and *Wolbachia*-negative cephalothoraxes (P=0.3630) despite *Pl. falciparum* only detected in *Wolbachia*-negative cephalothoraxes. However, *Wolbachia*-positive abdomens were associated with a lower sporozoites rate compared to *Wolbachia*-negative abdomens (P=0.0329). *16S rRNA* amplicon sequencing revealed no significant difference in alpha/beta diversities between abdomens and cephalothoraxes but the cephalothorax microbiome composition between *Wolbachia*-positive and *Wolbachia*-negative was significantly different (P<0.05).

**Conclusions:**

Our findings indicate a significant effect of the *w*AnD strain on the cephalothorax microbiome and potentially the ability of sporozoites to reach Salivary glands in mosquitoes with *Wolbachia*-infected abdomens. Further studies are needed to determine the mechanisms in which the *w*AnD strain interacts with *Plasmodium* sporozoites in *An. demeilloni* and if this strain could be used for malaria biocontrol through transinfection of major malaria vectors.

## Introduction

Vector-borne diseases (VBDs) contribute significantly to the global burden of mortality with an estimated 80% of the world’s population at risk of being infected by one or more vector-borne pathogens (WHO, 2017). VBDs include malaria, dengue, lymphatic filariasis, chikungunya, Zika and yellow fever which disproportionally affect tropical and subtropical areas. Malaria is the most important disease in terms of mortality, particularly in the WHO African Region, which often lacks the infrastructure to prevent, diagnose and treat malaria. Despite encouraging progress in vaccine development, insecticide resistance and mosquito behavioural changes continue to pose a threat to the effectiveness of frontline vector control tools including insecticide-treated bednets (ITNs) and indoor residual spraying (IRS) ( Gleave et al. 2025). ITNs and IRS were previously able to contribute to a significantly decrease of malaria transmission. However, during the last decade, progress has levelled off and the 2020 Global Technical Strategy milestones of reducing malaria morbidity by 75% and mortality by 90% in 2025 and 2030 respectively is now out of reach ( Barreaux et al. 2017). Rapid urbanisation, climate change and pollution have resulted in changing mosquito vector distributions such as the ongoing invasion of the urban malaria vector *An. stephensi* in the WHO African Region ( Sinka et al. 2020). Even newly developed insecticides for mosquito vector control will likely result in mosquito adaptation and current strategies using insecticides are failing given the rapid spread of resistance and logistical challenges of continued distribution via ITNs or IRS campaigns.

A potentially eco-friendly alternative strategy are bacteria that naturally reside within mosquitoes which have been shown to inhibit human pathogens (including *Plasmodium* malaria parasites). The major hurdle has been the lack of a natural mechanism to both spread bacteria through mosquito populations and allow for sustained high prevalence rates through maternal (vertical) transmission. An exception is *Wolbachia,* a bacterial endosymbiont that can invade mosquito populations through a reproductive phenotype called cytoplasmic incompatibility (CI), which results from the sterility of progeny from matings between *Wolbachia*-infected males and uninfected females. In *Aedes (Ae.) aegypti,* a mosquito species that does not have a stably associated *Wolbachia* in natural populations, it was possible to successfully introduce *Wolbachia* strains that were able to invade wild populations ( Walker et al. 2011; Hoffmann et al. 2011). *Wolbachia* replacement strategies have been successful in reducing dengue incidence by 77% in a randomised controlled trial in Indonesia ( Utarini et al. 2021). The lines infected with *Wolbachia* have been released into 16 dengue-endemic countries with >13.5 million people estimated to be protected. In a different control strategy, *Wolbachia* is also being used to suppress *Aedes* mosquito populations using male releases (insect incompatible technique or IIT). In Singapore, a release of male *Wolbachia*-infected *Ae. Aegypti* was followed by a significant decrease of the mosquito population and a 45% protective efficacy in released areas ( Lim et al. 2025). *Wolbachia*-based biocontrol strategies in *Anopheles* malaria vectors have been limited. However, two *Wolbachia* strains have been successfully introduced into *An. stephensi* lab colonies and resulted in significant *Plasmodium* parasite inhibition and CI induction ( Liang et al. 2024; Bian et al. 2013). To date, associated mosquito fitness costs in *Anopheles* following the introduction of *Wolbachia* strains has prevented progression to field release trials. Furthermore, *Wolbachia* strains used so far had been isolated from *Aedes* or *Drosophila* fruit fly species whilst optimal candidate strains might need to be from within the *Anopheles* genera as this might result in fewer fitness effects due to better adaptation of the host genus.

Historically *Wolbachia* was thought to not occur naturally within wild *Anopheles* populations but several reports have now detected strains ( Baldini et al. 2014; Baldini et al. 2018; Gomes et al. 2017; Jeffries et al. 2018; Niang et al. 2018; Ayala et al. 2019). Although the majority of these studies have used only PCR-based detection methods, the *w*AnD and *w*AnM strains were confirmed to be in genuine endosymbiosis in *An. demeilloni* and *An. moucheti* respectively and strains which can be visualised in mosquito ovaries ( Walker et al. 2021). For the first time *Anopheles Wolbachia* genomes were also sequenced and analysed ( Quek et al. 2022). The *w*AnD and *w*AnM strains dominate the microbiome and illumina genome sequencing obtained genome depths and coverages comparable to those of other known *Wolbachia* strains in genuine endosymbiosis ( Walker et al. 2021). In contrast, there is comparatively little evidence for *Wolbachia* strains in species within the *An. gambiae* complex – *An. coluzzii* and *An. gambiae* – which showed exceedingly low sequencing depth against *Wolbachia* genomes, despite high sequencing depth against mosquito genomes ( Walker et al. 2021). High density, stable *Wolbachia* strains are considered a prerequisite for an effective *Wolbachia*-based malaria vector control strategy but there have been contrasting studies demonstrating variable effects on *Pl. falciparum* prevalence. *Wolbachia* strains within the *An. gambiae* complex, collectively known as *w*Anga, consistently are present at the threshold limit of PCR detection despite numerous studies demonstrating an inhibitory effect on *P. falciparum* ( Gomes et al. 2017; Shaw et al. 2016). Conversely, high density *w*AnM *Wolbachia* strains in *An. moucheti* in Cameroon showed no evidence of reducing *P. falciparum* ( Mouillaud et al. 2023) although this study did not differentiate sporozoite and oocyst stages and *Wolbachia* density would have been predominantly from ovaries given the *w*AnM strain has previously been shown to be maternally inherited ( Walker et al. 2021). The *w*AnD strain in *An. demeilloni* is also a high-density strain that is maternally transmitted and contains cytoplasmic incompatibility factor ( *cif* ) genes that underpin CI in insects ( Walker et al. 2021) and warrants further investigation for malaria biocontrol ( Gnankine and Dabiré 2024). These studies raise the question whether *Wolbachia*-mediated resistance to *Pl. falciparum* in natural *Anopheles* malaria vectors is dependent on the mosquito species or the resident *Wolbachia* strain (or potentially a combination of both). In this study, we set out to determine if the *w*AnD strain was present in somatic tissue of *An. demeilloni* and if present was correlated to *Pl. falciparum* infective sporozoite stages and analysed the mosquito microbiome from adult females collected from diverse locations in eastern DRC.

## Methods

### Mosquito collection and species identification

**Figure 1.**
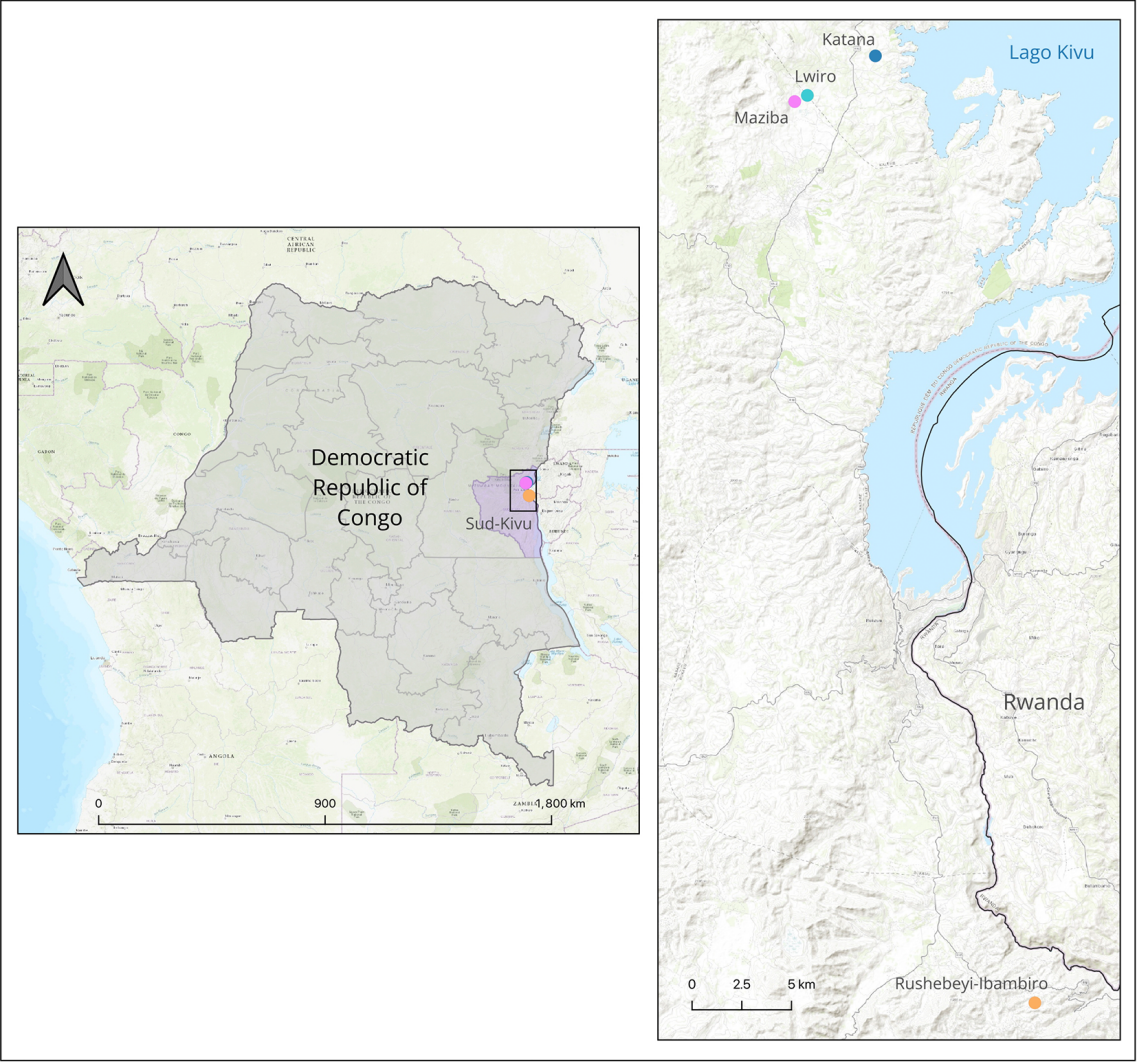
Map of Democratic Republic of the Congo (DRC) and the localisation of mosquito collection sites in the Sud-Kivu region of eastern DRC. Map was generated using QGIS 3.30 (Free Software Foundation Inc., Boston, MA, USA) using freely available administrative boundaries and inset map using a basemap from ESRI (Redlands, CA, USA).

Adult *Anopheles* mosquitoes were collected during the rainy season in four rural locations (Katana, Rushebeyi, Lwiro and Maziba) in Sud Kivu in the Democratic Republic of the Congo (DRC) in November 2021 and January-February 2024 ( Figure 1). Collections were undertaken using CDC light traps and morphological identification performed at the Centre de Recherche en Sciences Naturelles (CRSN), Lwiro, DRC using identification keys ( Coetzee 2020). Individual adult mosquitoes identified as *An. demeilloni* were stored in 1.5 mL Eppendorf tubes containing cotton pads and silica gel to prevent microbial contamination and transferred to the University of Warwick (UK) for further analysis. Adult female mosquitoes were subject to dissection to separate abdomens (containing ovaries) from the cephalothorax (containing Salivary glands) to allow a more accurate assessment of *Wolbachia* and *Plasmodium* sporozoites prevalence rates. Dissected body parts were individually placed in 96-well extraction plates and homogenised using a Qiagen Tissue Lyser II and Qiagen 5mm stainless beads. DNA was extracted using Qiagen DNeasy Blood and Tissue Extraction kit following manufacturer’s instructions. Extracted DNA was eluted in a volume of 50 μL and concentration quantified using Invitrogen Qubit DNA High Sensitivity Assay kits in combination with an Invitrogen Qubit 4 Fluorometer. Molecular identification of species was performed using a *An. demeilloni* specific qPCR assay targeting internal transcribed spacer (ITS2) sequence ( Walker et al. 2021). qPCR reactions were run on an Agilent Technologies Strategene Mx3005P in a final reaction volume of 10 μL containing 5 μL of Applied Biosystems™ SYBR™ Select Master Mix (catalogue no. 4472908), 1μL of 10X for each of the forward and reverse primers, 1μL of water and 2 μL of the extracted DNA. Reactions were run at 50°C for 2 minutes, 95°C for 2 minutes, followed by 40 cycles of 95°C for 15 seconds, 59°C for 22 seconds and 72°C for 1 minute. A melting cycle of 95°C for 10 seconds, 65°C for 60 seconds and 97°C for 1 second was run to ensure the correct target was amplified.

### *Wolbachia* detection and quantification

The detection and quantification of *Wolbachia* strain *w*AnD were undertaken on abdomens and cephalothorax samples using qPCR assays targeting the conserved *Wolbachia16S rRNA* gene ( Gomes et al. 2017). Each sample and no template controls (NTCs) were run in triplicate in a final reaction volume of 10 uL that consisted of 5 uL of Applied Biosystems™ SYBR™ Select Master Mix (catalogue no. 4472908) with a final concentration of 1 μM of each primer, 1 μL of water and 2 μL of the DNA samples. The qPCR assays were carried out in the Agilent Technologies Strategene Mx3005P under the following conditions: 50°C for 2 minutes, 95°C for 2 minutes, followed by 40 cycles of 95°C for 15 seconds, 57°C for 22 seconds and 72°C for 1 minute. A melting cycle of 95°C for 10 seconds, 65°C for 60 seconds and 97°C for 1 second was run to ensure the correct target was amplified. *Wolbachia* 16S rRNA gene copies per nanogram of total DNA was calculated from a standard curve of a synthetic oligonucleotide standard (Integrated DNA Technologies) used to calculate *16S rRNA* gene copies per ul after ten-fold serial dilutions ( Jeffries et al. 2021). The qPCRs results were analysed using Agilent Technologies software.

### Sanger sequencing of the *wsp* gene

*Wolbachia* surface protein ( *wsp*) gene sequence analysis were carried out using primers wsp81F: 5’-TGGTCCAATAAGTGATGAAGAAAC-3’ and wsp691R: 5’-AAAAATTAAACGCTACTCCA-3’ ( Zhou et al. 1998) in a Bio-Rad T100 Thermal Cycler using previously optimised cycling conditions (Jeffries et al. 2018). The PCR reactions were optimised in a final volume of 20 μL containing 10 μL of 2X Phire Hot Start II PCR Master Mix (Thermo Scientific, USA), 2 μL of 10X for each primer, 4 μL of water and 2 μL of DNA. PCR products were separated and visualised using 2% E-Gel EX agarose gels (Invitrogen) with SYBR safe and an Invitrogen E-Gel iBase Real-Time Transilluminator. PCR products were submitted to Source BioScience (Source BioScience Plc, Nottingham, UK) for PCR reaction clean-up, followed by Sanger sequencing to generate forward reads. Sequencing analysis was carried out in 4peaks ( https://nucleobytes.com/4peaks/) and consisted of sequences being manually checked, edited, and trimmed as required. Sequences were used to perform NCBI BLAST database queries and searches against the *Wolbachia* PubMLST database ( Jolley et al. 2018) for strain typing. Sequence alignments were constructed in MEGA 12 ( Kumar et al. 2024) ( https://www.megasoftware.net) using the Muscle algorithm to include relevant sequences from the BLAST analysis and *Wolbachia* MLST database. The phylogenetic tree was generated using the Maximum Likelihood method based on the Tamura-Nei model of nucleotide substitutions. The tree with the highest log likelihood after 1000 bootstrap replicates was shown. The percentage of trees in which the associated taxa clustered together is shown below the branches.

### *Plasmodium falciparum* detection

*Plasmodium falciparum* detection on mosquito samples was performed using a qPCR assay targeting the *Pl. falciparum* sporozoite cytochrome c oxidase subunit 1 ( *Cox1*) mitochondrial gene ( Marie et al. 2013). The qPCR reactions were prepared and optimised using 5 mL of Agilent Brilliant III Ultra-Fast SYBR Green Low ROX qPCR Master Mix (catalogue no. 600892) with 1 mL for each primer (5′-TTACATCAGGAATGTTATTGC-3′ and 5′-ATATTGGATCTCCTGCAAAT-3′) with a final concentration of 1mM of each primer, 1mL of water and 2mL of DNA. Each sample and NTCs were run in triplicate in the Agilent Technologies Strategene Mx3005P using the following cycling conditions: 95°C for 3 minutes, followed by 40 cycles of 95°C for 20 seconds, 60°C for 22 seconds and 72°C for 1 minute. A melting cycle of 95°C for 10 seconds, 65°C for 60 seconds and 97°C for 1 second was run to ensure the correct target was amplified.

### Microbiome analysis

A sub-sample of DNA from the same individual mosquito for both the abdomen and corresponding cephalothorax underwent Illumina amplicon sequencing of the *16S rRNA* gene including variable *Wolbachia* infection status (based on qPCR). Illumina sequencing targeted the V1-V2 hypervariable regions of the *16S rRNA* gene using the universal primers 27F (5’-ACACTCTTTCCCTACACGACGCTCTTCCGATCTNNNNNAGAGTTTGATCMTGGCTCAG-3’) and 338R (5’-GTGACTGGAGTTCAGACGTGTGCTCTTCCGATCTTGCTGCCTCCCGTAGGAG- 3’). Libraries were prepared and sequenced by the Genomics Facility at the School of Life Sciences, University of Warwick (UK). Amplicon sequencing was achieved using Illumina MiSeq v3 (Illumina, USA) and resulting data received as FASTQ files of the demultiplexed paired end reads. All analysis of resulting reads was carried out in R software version 4.4.0 ( *www.R-Project.Org/* ). FASTQ files were then analysed using a DADA2 pipeline ( Callahan et al. 2016) after primer sequences were removed using the Cutadapt package ( Martin 2011). Samples with fewer than 1000 reads per sample were removed from the dataset. Read quality profiles were visualised with a quality score of 20 accepted as a minimum. Reads were then filtered and trimmed based on the observed quality. Paired-end reads were merged and concatenated due to the non-overlapping nature of the V1-V2 hypervariable regions. Taxonomy was assigned using the Silva database (Version 138.1 SSU), and the resulting taxonomic assignments were then visualised and explored using the Phyloseq package ( McMurdie and Holmes 2013).

### Statistical analysis

Comparative statistics were carried out using the JMP 18.0 software (SAS Institute. Inc, North Carolina). General Linear Models were performed assuming a binomial distribution of *Wolbachia* infection in body parts and across different locations where mosquitoes were sampled. The interaction between both variables was tested but removed from the model if not significant. *Wolbachia* density across the different body parts was compared using a Mann-Whitney test and a Spearman rank correlation coefficient was performed to compare *Wolbachia* density between the abdomen and the cephalothorax. Logistic regressions were performed to test the effect *Wolbachia* infections in the likelihood of *Plasmodium* infection in mosquitoes. A power analysis was performed using JMP data analysis software ( https://www.jmp.com/en/software/data-analysis-software) to determine the minimum sample size to detect a difference of *Plasmodium* infection rates between *Wolbachia* positive and negative samples based on the two-sided test of two independent proportions with a significance level of 0.05 and a power of 0.80. Microbiome diversity was explored by assessing alpha and beta diversity. Alpha diversity was measured using both Simpson and Shannon diversity indices, and beta diversity measured using Bray-Curtis measure of dissimilarity. The difference between the microbiome composition in DNA extracted from abdomen vs cephalothorax was assessed using permutational analysis of variance (PERMANOVA) analysis using the vegan package ( Oksanen 2025). The composition of the microbiome was observed using relative abundance of data agglomerated to the genus level.

## Results

### *Wolbachia w*AnD strain is found at significantly higher prevalence rates in abdomens compared to cephalothoraxes of *An. demeilloni*

**Figure 2.**
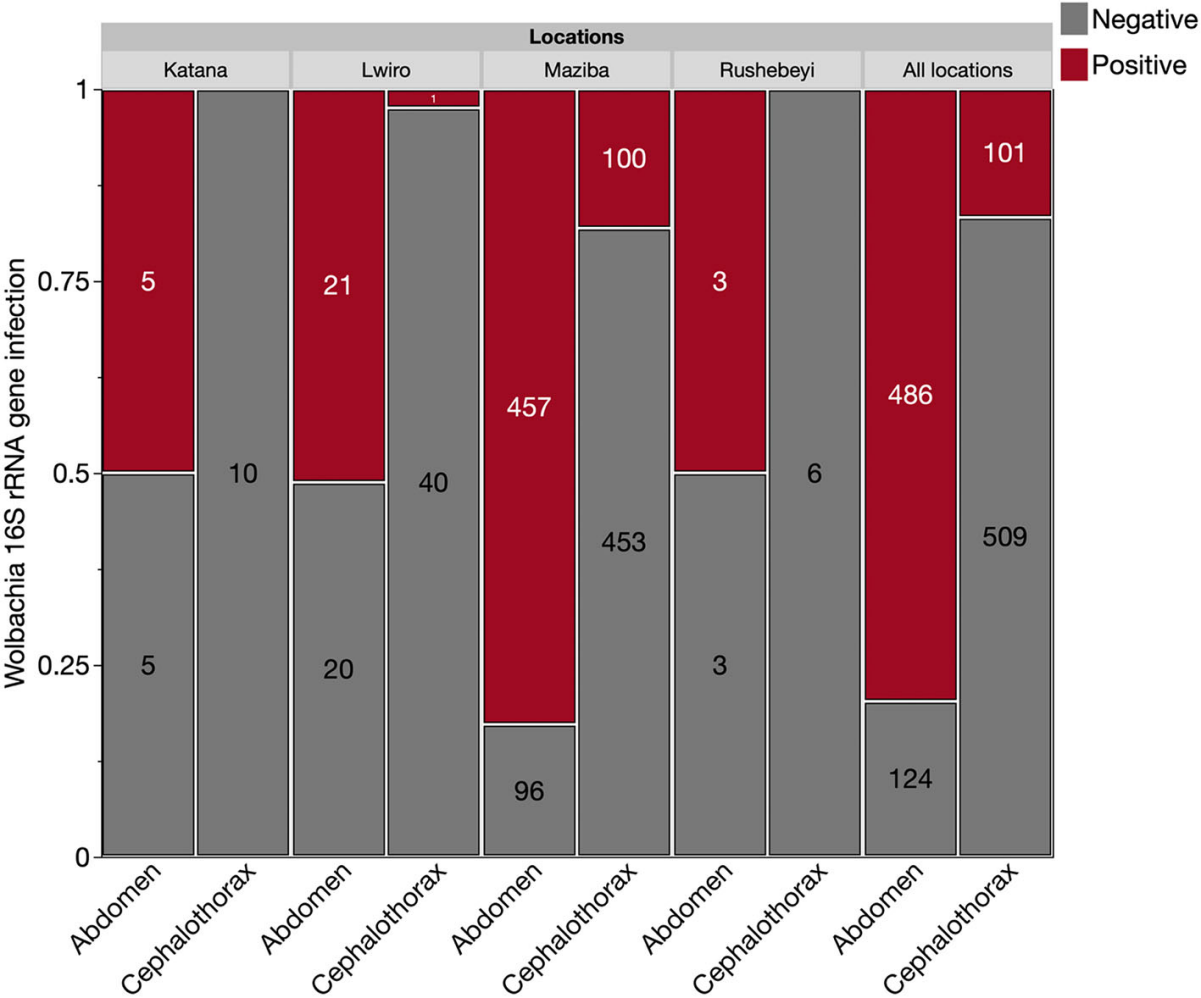
*Wolbachia* prevalence rates in the abdomens and cephalothorax of *An. demeilloni* individuals collected in 2021 in the four regions of the DRC.

**Figure 3.**
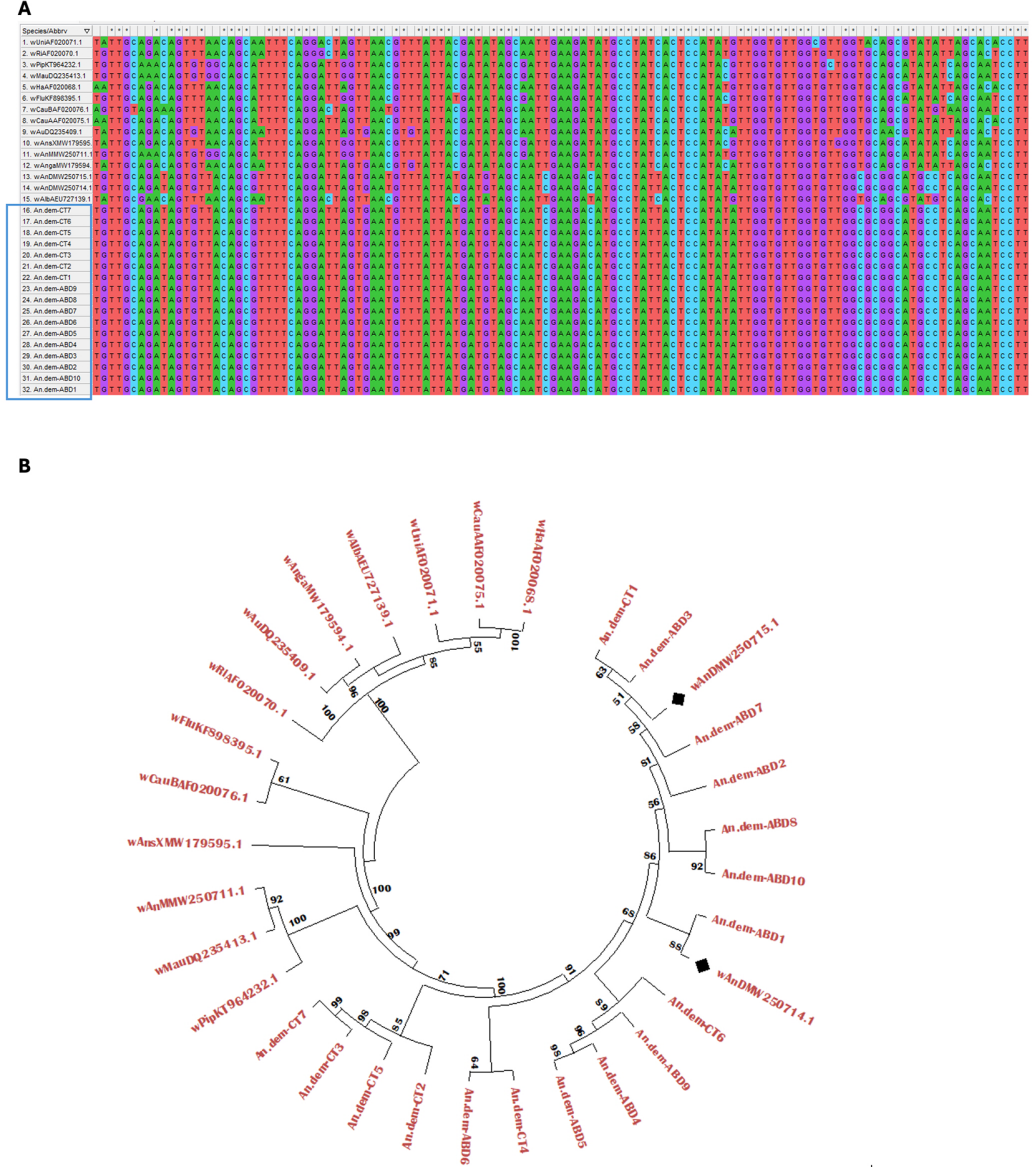
*Wolbachia* surface protein (wsp) gene analysis from *An. demeilloni* abdomens and cephalothoraxes. **A)** Multi-sequence alignment of wsp sequences from our study and reference sequences, AB = abdomen, CT = cephalothorax. **B)** NCBI accession numbers are provided for reference strains, blue box indicates our study sequences alongside two *w*AnD strain *wsp* references. The phylogeny was inferred generated Mega 12 using the Maximum Likelihood method and Tamura-Nei (1993) model ( Tamura and Nei 1993) of nucleotide substitutions and the tree with the highest log likelihood (-3,062.04) is shown. The percentage of replicate trees in which the associated taxa clustered together (1,000 replicates) is shown below the branches ( Felsenstein 1985). The initial tree for the heuristic search was selected by choosing the tree with the superior log-likelihood between a Neighbour-Joining (NJ) tree ( Saitou and Nei, 1987) and a Maximum Parsimony (MP) tree. The NJ tree was generated using a matrix of pairwise distances computed using the Tamura-Nei (1993) model ( Tamura and Nei 1993). The MP tree had the shortest length among 10 MP tree searches, each performed with a randomly generated starting tree. The analytical procedure encompassed 32 nucleotide sequences with 677 positions in the final dataset. Evolutionary analyses were conducted in MEGA12 ( Kumar et al. 2024) utilizing up to 4 parallel computing threads.

A total of 672 mosquitoes morphologically identified as *An. demeilloni* were analysed from collections in 2021 from four different areas in eastern DRC (Maziba, Katana, Lwiro and Rushebeyi) in addition to a further 96 mosquitoes collected in 2024 from Lwiro. As other *Anopheles* species are present in DRC including *An. funestus* which is morphologically similar to *An. demeilloni*, we first confirmed species using a qPCR assay previously developed to specifically target the ITS2 region of *An. demeilloni* ( Walker et al. 2021). Using this assay, the accuracy of morphological identification was calculated at 91.7% (704/768). We then determined *Wolbachia* prevalence rates in *An. demeilloni* samples using a qPCR assay targeting the conserved *Wolbachia 16S rRNA* gene. A total of 610 individual *An. demeilloni* females collected in 2021 were analysed according to body part (cephalothorax and abdomen) to provide a more comprehensive assessment of relative infection rates given mosquito abdomens contain ovaries which are the primary tissues for *Wolbachia* infection due to maternal transmission. The corresponding cephalothoraxes provided a measure of *Wolbachia* somatic tissue infection and is often used as a proxy for Salivary glands. Overall, the *Wolbachia* prevalence rate was significantly higher in abdomens (80%) compared to the cephalothoraxes (17%) (χ ^2^ = 1066.86 Df = 1 *P<0.0001*) ( Figure 2). There was also a significant difference in *Wolbachia* prevalence rates across geographical areas, with significantly higher prevalence rates observed in abdomens of individuals collected in Maziba compared to their counterparts in other areas (χ ^2^ = 39.82 Df = 3 *P < 0.0001*) ( Figure 2). Interestingly, somatic *Wolbachia* infections in the cephalothoraxes of *An. demeilloni* were detected in 18% and 2% respectively in Maziba and Lwiro. However, our analysis provided no evidence of *Wolbachia* infection in the cephalothoraxes of females collected in Katana and Rushebeyi. To provide stronger evidence of somatic infection, we undertook sanger sequencing of the *Wolbachia* surface protein ( *wsp*) gene on paired *An. demeilloni* cephalothoraxes and abdomens shown to be *Wolbachia*-positive through *16S rRNA* qPCR. All our sequences (available at https://osf.io/y6sh8) clustered with existing *w*AnD *wsp* sequences, accession numbers in GenBank MW250715.1 and MW250714.1 in BLAST analysis and phylogenetic analysis ( Figure 3A, 3B) indicating no *w*AnD strain variation was detected.

Quantitative PCR was used targeting a fragment of the conserved *Wolbachia 16S rRNA* gene with samples run in triplicate alongside standard curves and no template controls (NTCs). Values in the mosaic plot represent the number of individuals in each category based on *Wolbachia w*AnD strain infection status.

### *Wolbachia* densities are higher in abdomens compared to cephalothoraxes

**Figure 4.**
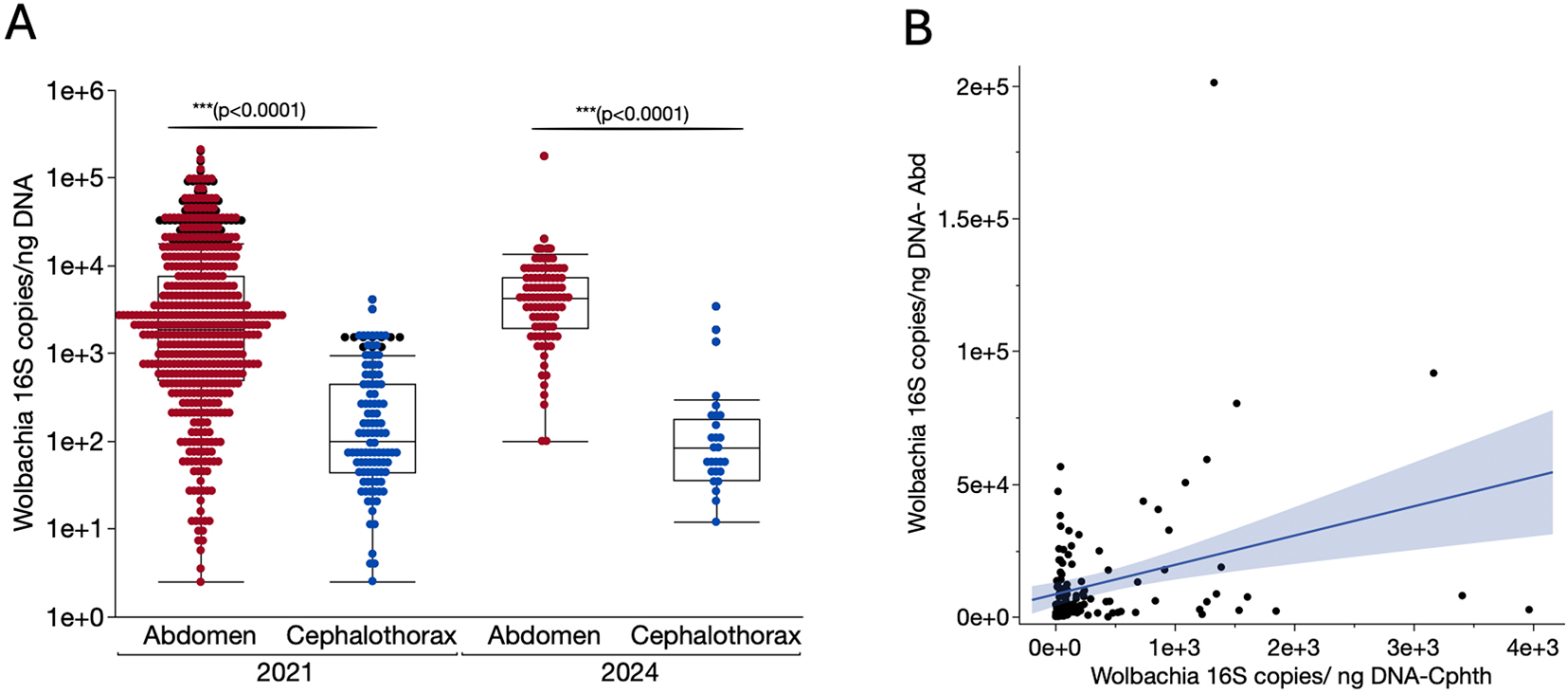
*Wolbachia* density in abdomens and cephalothoraxes of *An. demeilloni.* **A.** Scatter plot combined with box plot generated for normalized *Wolbachia w*AnD strain densities ( *16S rRNA* gene copies/ng DNA) quantified through qPCR of the *Wolbachia* using a standard curve generated after 10-fold serial dilutions of a synthetic oligonucleotide standard. The boxes represent the 25 and 75 percentiles while the whiskers indicate maximum and minimum values. The median is depicted as the horizontal line. A total of 672 and 96 individual *An. demeilloni* females collected in 2021 and 2024 respectively were dissected and *Wolbachia* densities determined by qPCR. **B**. Scatter plot showing the relationship between *Wolbachia 16S rRNA* gene copies in the abdomens and the corresponding cephalothoraxes. The line of fit in blue was determined using linear regression, *r _s_* = 0.2531, *P = 0.0046.*

We then assessed *Wolbachia* densities by compared normalized levels of *Wolbachia* infection in the different body parts using a synthetic oligonucleotide standard and considering total DNA present in the qPCR reactions. A Mann-Whitney test indicated (as expected) significantly higher *Wolbachia* densities were present in the abdomens compared to cephalothoraxes ( *P < 0.0001*) ( Figure 4A). The mean normalised *Wolbachia* density (copies/ng DNA) in abdomens was 8.23 × 10 ^3^ (±1.81 ^4^) compared to 3.63 × 10 ^2^ (±6.18 × 10 ^2^) for cephalothoraxes. This pattern did not significantly vary across years, and the level of infection remained higher in the abdomen 6.79 × 10 ^3^ (±1.83 ^4^) compared to the cephalothorax 3.25 × 10 ^2^ (±7.42 × 10 ^2^) ( *P < 0.0001*) in samples collected in 2024. A Spearman’s rank correlation analysis between *Wolbachia* densities calculated per nanogram of the total DNA in the cephalothorax and the corresponding abdomens revealed a significant positive correlation ( *r _s_* = 0.2531, *P = 0.0046*, Figure 4B) indicating a high *Wolbachia* density in the abdomen could result in high densities in the corresponding cephalothorax. In addition, high density of *Wolbachia* infection in the abdomen was found in samples with *Wolbachia*-positives cephalothorax compared to their negative counterparts ( *P < 0.0001*).

### Co-infection rates of *Plasmodium falciparum* and *Wolbachia* in *Anopheles demeilloni*

**Figure 5.**
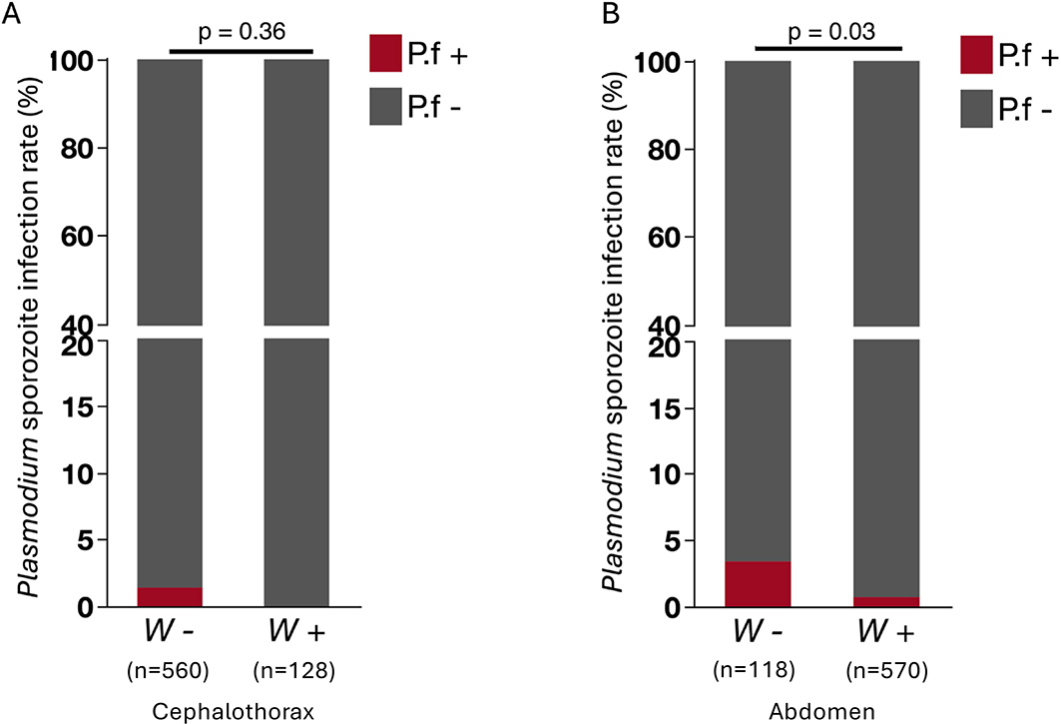
Correlation between *Pl. falciparum* sporozoites rates (in cephalothoraxes) and *Wolbachia* strain *w*AnD in *An. demeilloni.* **A)** in the cephalothoraxes and **B)** in the abdomens. Quantitative PCR targeting the *Cox1* gene of *Pl. falciparum* was used to screen positive and negative- *Wolbachia* cephalothorax and abdomens run in triplicate alongside standard curves and no template controls (NTCs). *Pl. falciparum-*infected mosquitoes were only found in *Wolbachia*-negative cephalothoraxes (8/560) and not in *Wolbachia*-positive cephalothoraxes (0/120).

We determined whether malaria parasites reached the infectious sporozoite stage in wild *An. demeilloni* from our collection locations in the DRC by screening cephalothoraxes (a proxy for Salivary gland stage sporozoite infection). An overall prevalence rate of 1.3% (9/704) was found in all *An. demeilloni* samples. We then investigated whether the presence of *Wolbachia w*AnD correlated with the presence of *Pl. falciparum* in cephalothoraxes using only samples from Maziba and Lwiro that showed positive *Wolbachia* infections in their cephalothorax. The prevalence rate of *Pl. falciparum* was not significantly different between *Wolbachia*-positive individuals compared to *Wolbachia*-negative individuals ( *P = 0.3630*). However, interestingly all *Pl. falciparum-*infected mosquitoes were only found in *Wolbachia*-negative cephalothoraxes (8/560) ( Figure 5A). A power analysis performed showed a minimum sample size of 1106 mosquitoes would affect the development of *Plasmodium* sporozoites, from 1.4% in *Wolbachia-*negative individuals to below the limit of detection in *Wolbachia*-positive mosquitoes at a level of 0.05 and a power of 0.80. A total of 688 mosquitoes was analysed in our study, which is below the sample size required to detect a significant effect of *Wolbachia* affecting *Pl. falciparum.* We then investigated whether the presence of *Wolbachia w*AnD in the abdomen had any correlation to the presence of *Pl. falciparum* in the corresponding cephalothoraxes ( Figure 5B). We found significant difference in the prevalence rates of *Pl. falciparum* between individuals with *Wolbachia* abdomen positive (4/570) and individuals with *Wolbachia* abdomen negative (4/118) ( *P = 0.0329*).

### *Anopheles demeilloni* microbiome composition and diversity

**Figure 6.**
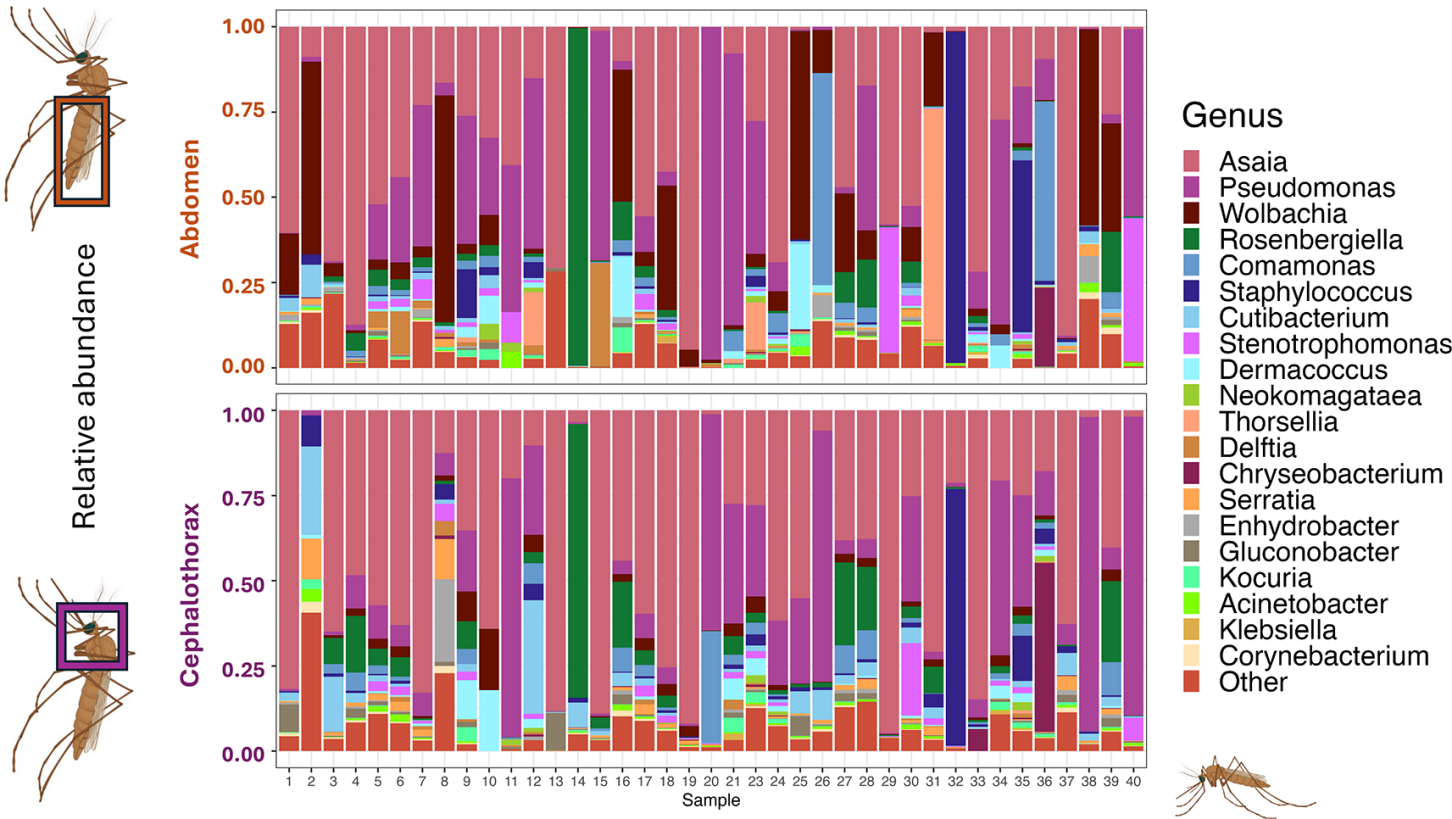
Relative abundance of the top 20 most abundant bacterial genera identified in 40 *An. demeilloni* individuals. **Data are separated into abdomens (upper barplots) and corresponding cephalothoraxes (lower barplots). Genera are listed in order of highest overall abundance. “Other” represents the total of all genera that were not in the top 20 most abundant. Mosquito images created in** **biorender.com** . Individual sample numbers are provided to allow comparison between body parts of individuals.

**Figure 7.**
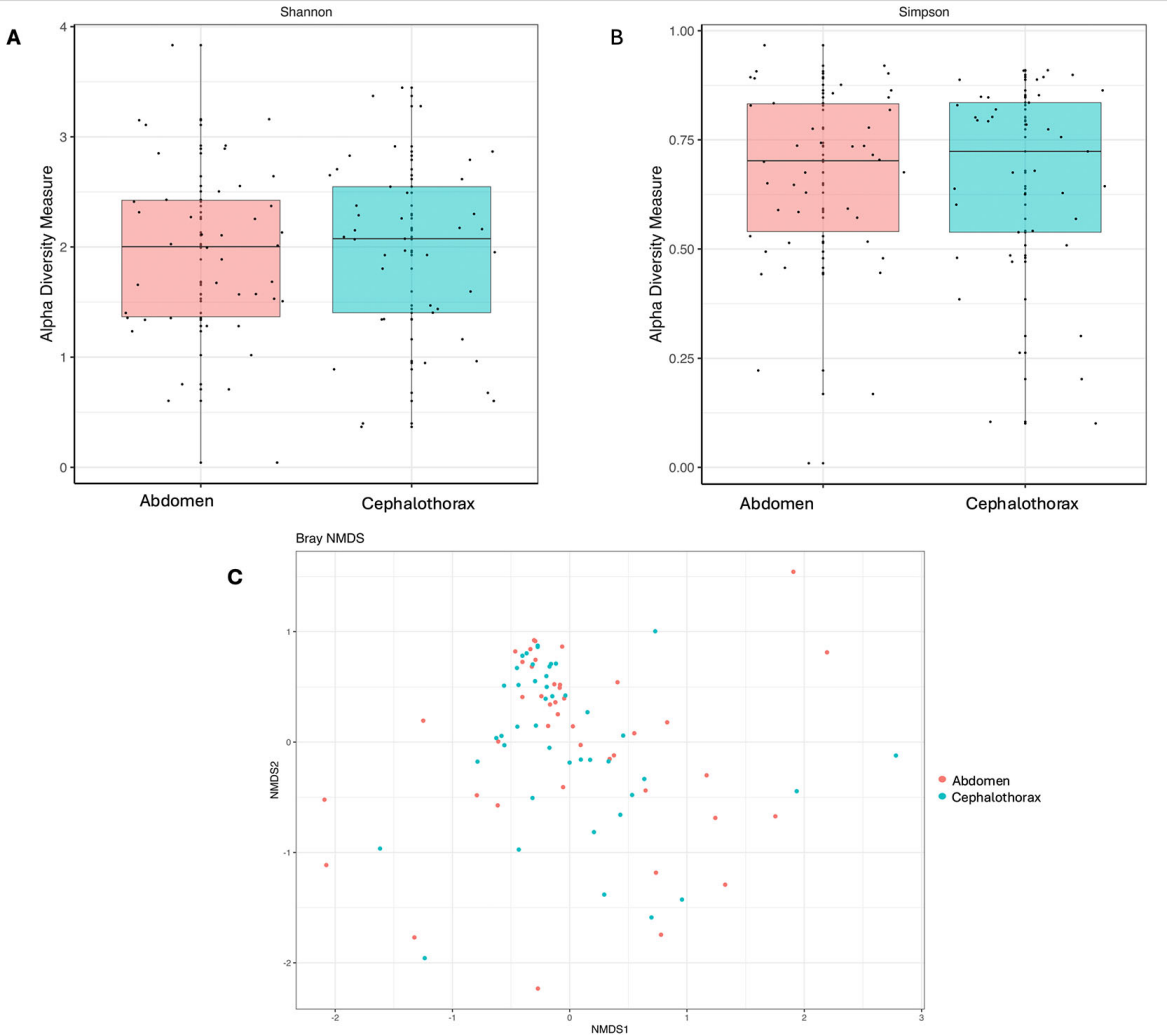
Microbiome diversity of *An. demeilloni* abdomens and cephalothoraxes. Alpha diversity assessed using **A)** Shannon’s diversity index and **B)** Simpson’s diversity index. In both panels the central line represents the median of the data set, with the upper and lower limits of the boxplot representing the upper and lower quartiles, vertical lines extend to encompass data points within 1.5 times the calculated inter-quartile range. There were no data points that exceeded this. **C)** Beta diversity for the microbiome of *An. demeilloni* abdomens and cephalothoraxes assessed using Bray-Curtis measure of dissimilarity. All *Wolbachia*-assigned reads were removed from the datasets before analysing alpha and beta diversity.

Following Illumina *16S rRNA* amplicon sequencing, all samples with fewer than 1000 resulting reads were removed from the dataset, leaving a total of 42 abdomen and 41 cephalothoraxes for further analysis. *Wolbachia* was identified in abdomens and cephalothoraxes at prevalence rates of 76% and 54% respectively ( Figure 6). As expected, there were no cephalothorax samples positive for *Wolbachia* for which the corresponding abdomen was not positive. *Wolbachia* was also found in higher relative abundances in abdomen samples than cephalothorax. For diversity analysis, all *Wolbachia*-assigned reads within the dataset were removed to assess the diversity within the remaining microbiome community given when present *Wolbachia* can dominate the microbiome. Overall alpha diversity, assessed using the Shannon’s and Simpson’s diversity indices, showed no significant difference between body parts ( Figure 7A,B). Bray-Curtis dissimilarity analysis was used to assess the beta diversity and there was no distinct grouping suggesting no significant difference in the microbiome composition between mosquito body parts despite a high level of inter-individual differences ( Figure 7C). Following the removal of all *Wolbachia*-assigned reads within the dataset, PERMANOVA analysis was used to assess the difference of microbiome composition between sample body parts and *Wolbachia* infection status. There was no significant difference between the composition of the abdomen and cephalothorax microbiome (F(1,82) = 2.136, P = 0.144). There was also no significant difference between *Wolbachia*-positive and *Wolbachia*-negative abdomen samples (F(1,40) = 0.138, P = 0.704). However, there was a significant difference in the composition of the microbiome between *Wolbachia*-positive and *Wolbachia*-negative cephalothorax samples (F(1,39) = 4.0305, P < 0.05).

## Discussion

The *w*AnD *Wolbachia* strain in *An. demeilloni* was previously shown to result in high density endosymbiotic associations ( Walker et al. 2021) and in this study we aimed to determine if this strain was 1) present in somatic tissues and 2) if it correlates to the presence of *Pl. falciparum* infective stage sporozoites and impacts the overall mosquito microbiome. When analysing these associations, we included molecular species identification using species-specific qPCR analysis to demonstrate morphological identification accuracy of 92%. Although previous studies have correlated the presence of *Wolbachia* with *Pl. falciparum* in other wild *Anopheles* mosquito species ( Gomes et al. 2017; Shaw et al. 2016; Mouillaud et al. 2023), our study aimed to remove the confounding factor that *Wolbachia* is maternally transmitted and heavily infects the ovaries resulting in erroneous correlations obtained from analysing whole mosquito bodies. Dissected body parts also allowed isolated detection of sporozoites as Salivary glands are the only tissue within the cephalothorax, to our knowledge, in which infective sporozoite stages are found. Our results demonstrated no evidence of *Wolbachia/Plasmodium* co-infections in cephalothoraxes but the presence of the *w*AnD strain was not statistically correlated with lower levels of *Pl. falciparum.* The low *P. falciparum* infection rate (1.4%) prevented a statistically significant result based on our power calculations. However, we did find a statistically significant difference in the sporozoite rates between *Wolbachia*-infected/uninfected abdomens suggesting the possibility that the *w*AnD strain could potentially reduce *Plasmodium* sporozoites. We also found a higher level of *w*AnD in the abdomen of individuals with *Wolbachia*-positive cephalothorax compared to *Wolbachia*-negative cephalothoraxes.

Our study is limited by analysis of wild caught mosquitoes which prevents the inclusion of controls to fully elucidate any direct association between the *w*AnD strain and *Plasmodium.* Collection of wild *Anopheles* mosquito populations does not ensure that all individuals females are old enough to ensure that vertically transmitted *Wolbachia* bacteria such as the *w*AnD strain have replicated and colonized host tissues such as Salivary glands. Age-dependent *Wolbachia* densities are observed in other mosquito species such as *Ae. albopictus* ( Tortosa et al. 2010). Furthermore, mosquito age at the point of collection also influences sporogony so that only older females have lived long enough for *Pl. falciparum* sporozoites to have reached Salivary glands. Despite these limitations, our results align with those reported in *An. gambiae* complex species from Mali in which a negative correlation between *Wolbachia*-infected mosquitoes ( *w*Anga-Mali strain) and *Pl. falciparum* was reported ( Gomes et al. 2017). High *w*Anga prevalence rates in samples encompassing both abdomens and thoraxes were also correlated to low *Pl. falciparum* prevalence rate in five-day post-collections of blood-fed *An. coluzzii* in Burkina Faso ( Shaw et al. 2016). Colonization of *An. demeilloni* would provide the opportunity to undertake laboratory vector competence experiments. *Plasmodium* inhibition was demonstrated after successful transinfection of *An. stephensi* colonies with the *w*AlbB and *w*Pip strains of *Wolbachia* ( Liang et al. 2024; Bian et al. 2013) despite both strains having no impact on arbovirus transmission in their native mosquito hosts.

We previously showed that when present in whole body adult females, *w*AnD is the dominant bacterial species in *An. demeilloni* ( Walker et al. 2021) but when extending this to just the cephalothorax we do not see this same microbiome dominance. However, caution must be taken in using ‘association’ studies from wild mosquitoes as there are numerous environmental factors including acquisition of environmental bacterial species such as *Asaia* that contribute to the microbiome in collection locations. Although there has been an antagonistic relationship between *Wolbachia* and *Asaia* observed in *An. gambiae* and *An. stephensi* ( Hughes et al. 2014; Rossi et al. 2015), further studies have shown evidence of coinfections in members of the *An. gambiae* complex in Guinea ( Jeffries et al. 2021) and more studies are showing that this relationship is dependent on the species/strain of mosquitoes, geographic location of collection and tissue localisation ( Ilbeigi Khamseh Nejad et al. 2024).

Our analysis included *An. demeilloni* samples from diverse collection locations in eastern DRC and we were able to detect high but variable prevalence rates. Variable prevalence rates in geographical distinct populations could result from genetic diversity within the host species, affecting the prevalence of *Wolbachia* infection in these mosquito populations. Variable *w*AnD prevalence could also be due to our study adhering to Minimal information for Publication of Quantitative Real-Time PCR Experiments (MIQE) established standards which increases the possibility of ‘false negative’ low-density infections. Sequencing of the *wsp* gene indicated no variability in *Wolbachia* strain typing and confirmed genuine *w*AnD strain infections. The presence of the *w*AnD strain in Maziba at high prevalence rates provides further evidence of the ability of this strain to ‘invade’ populations and this is likely due to induction of CI. We previously demonstrated maternal transmission of the *w*AnD strain ( Walker et al. 2021) but we have now shown for the first time that this strain can infect somatic tissue (in the cephalothorax). Tissue-specific infection should be further investigated through dissection of Salivary glands and the use of multiple detection methods including fluorescence *in-situ* hybridization (FISH). *Wolbachia* strain tissue tropism can vary between mosquito species but somatic infection is consistent with high density strains that impact human pathogens ( Walker et al. 2011; Liang et al. 2024; Bian et al. 2013; Joubert et al. 2016). Transient *Wolbachia* infections in *An. gambiae* also indicate the potential to actively colonise somatic tissue and inhibit the development of *Plasmodium* ( Hughes et al. 2011). *Wolbachia*’s inhibitory effects on *Plasmodium* parasites has previously been shown to be associated with upregulated of immune genes in *An. gambiae* after intrathoracic inoculation ( Kambris et al. 2010). Exciting new avenues to investigate will be determining if the presence of the *w*AnD strain is priming the basal immune system in the abdomen and to characterise the molecular mechanisms of *Wolbachia-Plasmodium* interactions in *An. demeilloni* colonies to determine any direct evidence of inhibition. Currently there are limited studies on *An. demeilloni* as it is not assumed to be a major malaria vector in the WHO African Region. In eastern DRC, it has been collected alongside the main malaria vectors *An. gambiae* s.l and *An. funestus* (Jeffries et al. 2018; Walker et al. 2021) which have been much more extensively studies and show high malaria prevalence rates (Jeffries et al. 2018). Currently there is limited evidence to date to determine if the presence of the *w*AnD strain could be a contributing factor to the status of *An. demeilloni* as a presumed non malaria vector.

## Conclusions

This study has demonstrated that there are high but variable prevalence rates of the wAnD strain in *An. demeilloni* in Eastern DRC and this strain infects the mosquito cephalothorax and influences the overall microbiome. We detected no co-infections with *Pl. falciparum* sporozoites but found no statistical significance due to low sporozoite rates and limited sample numbers. There was a negative correlation of *Wolbachia*-infected abdomens and sporozoites in the cephalothorax adding to the potential of *Wolbachia* strains in preventing malaria transmission through transinfection of major malaria vector species. Further studies are needed – particularly with *An. demeilloni* lab colonies – to determine if there is pathogen inhibition and the mechanisms behind this interaction.

## Data availability

### Underlying data

Quantitative PCR data, additional microbiome analysis (with *Wolbachia* reads) and *wsp* FASTAs from Sanger sequencing are available at https://osf.io/y6sh8

Data are available under the terms of the Creative Commons Attribution 4.0 International license (CC-BY 4.0).
